# Tumour induction by methyl-nitroso-urea following preconceptional paternal contamination with plutonium-239

**DOI:** 10.1038/sj.bjc.6690403

**Published:** 1999-05-01


*British Journal of Cancer* (1999) **80**(3/4), 627–628
© 1999 Cancer Research Campaign
Article no. bjoc.1998.0403

Letter to the Editor

### Tumour induction by methyl-nitroso-urea following preconceptional paternal contamination with plutonium-239

**Sir**

We read with interest the paper by Lord et al (1998) (*Br J Cancer* **78**: 301–311). On the basis of their findings the authors state that ‘there are no grounds for suggesting that these results explain the Seascale phenomena’. On the other hand, the authors do appear to see a role for their observations in cancer induction in offspring in man, since they consider it ‘possible that … offspring of fathers similarly exposed, albeit at considerably lower dose levels, could also carry such an increased susceptibility to a secondary insult’. There are a number of features of their results, together with a number of other studies not discussed in the paper, which do not support this proposition.

One feature of their study (Table 6 and Figures 5 and 6) was that leukaemia and lymphoma rates were higher in methyl-nitroso-urea (MNU)-treated offspring of male mice injected with 128 Bq $g^{-1}$ of $^{239}Pu$ than in MNU-treated offspring of fathers injected with 256 Bq $g^{-1}$ of $^{239}Pu$, with the rates in both groups higher than in the MNU-treated offspring of carrier (unirradiated) animals. Lord et al (1998) argue that such a non-monotonic dose–response might be explicable by cell-sterilization effects of the sort observed in the UK ankylosing spondylitis patients (Smith and Doll, 1982; Weiss et al, 1995). However, this comparison is misleading. The spondylitics were a population *directly* exposed to ionizing radiation. It is difficult to see how the competing effects of cell-sterilization and mutation apply in the case of paternal preconception irradiation (PPI), where inactivation of a sperm cell would result in no offspring from that cell. The statistics given in Table 6 and Figures 5 and 6 are all conditional on there being offspring, so that the hypothesized cell-sterilization effect would disappear in relation to the end points considered there. Consequently, the absence of a monotonic dose–response for any of the measures given in Table 6 and Figures 5 and 6 argues against a causal association between these end points and PPI.

It is clear from Figure 6 that cumulative lymphoma yield is if anything *reduced* in the two paternally irradiated groups, although as pointed out by the authors lymphomas (but not leukaemias) occur significantly earlier in the two paternally exposed groups. This apparently paradoxical result is a function of the very high leukaemia/lymphoma cumulative incidence (which in all groups exceeded 70%); the main effect of PPI by $^{239}Pu$ is to apparently increase the proportion of leukaemias relative to lymphomas.

There appears to be a more convincing PPI dose–response for the chromosome aberration data. However, the elevation in chromosome aberration rate is only significant for bone-marrow cells, and not spleen cells; even for bone-marrow cells a statistically significant elevation in chromosome aberration rates (and a monotonic dose–response) is only observed for 2-day cultures, but not for 7-day cultures. Given the multiplicity of culture times and samples considered it is difficult to attach much significance to this finding.

There appears to have been no attempt to randomize parents between the two $^{239}Pu$-paternally irradiated and control groups. In the light of the very high tumour rates in the control group this makes the interpretation of the results of the study problematic. As discussed by Selby (1990), when the ‘spontaneous’ tumour rates are high, the observed differences between $^{239}Pu$-paternally irradiated and control groups may arise from segregation of pre-existing mutations in these groups. In addition, there does not appear to have been any attempt to assess tumour incidence in animals ‘blind’ to their exposure status, so that ascertainment bias may be present.

In the West Cumbria leukaemia and lymphoma case-control study, Gardner et al (1990) found a statistically significant association between relatively high external doses of radiation measured by film badges worn by men employed at the Sellafield nuclear installation before the conception of their children, and the incidence of leukaemia in these children. At the time it was suggested, for example by Beral (1990), that the external doses may have been a surrogate for doses received from internally deposited radionuclides. However, in a comprehensive study of cancer in the offspring of Sellafield workers, the Health and Safety Executive (HSE, 1993, 1994) found ‘an absence of any association with internal radiation dose’, including the dose from alpha-emitting radionuclides which was dominated by the dose from isotopes of plutonium. These internal doses were calculated by the National Radiological Protection Board from biological monitoring data held at Sellafield. Consequently, the association between PPI and childhood leukaemia in West Cumbria concerns external, rather than internal, dose.

The HSE study found that the association between PPI, as measured by film badges, and childhood leukaemia was confined to children born in the village of Seascale, and that there was ‘no indication of any association’ for children born outside Seascale (HSE, 1994). This is despite the fact that 92% of the children of the Sellafield workforce born in Cumbria were born outside Seascale and that 93% of the associated collective PPI dose was associated with non-Seascale births (Parker et al, 1993). The Seascale association of leukaemia with PPI is statistically strongly inconsistent with the lack of association found not only among children of the Sellafield workforce born elsewhere in West Cumbria, but also with the results of studies of the offspring of the Ontario and Scottish nuclear workforces and of the Japanese atomic bomb survivors (Little et al, 1994). The association of leukaemia with PPI in Seascale is also statistically inconsistent with the absence of such an association in the offspring of Danish neurosurgical patients who had received substantial testicular doses of alpha radiation as a result of the administration of the diagnostic contrast medium Thorotrast (Little et al, 1996). Recently, the record linkage study of cancer in the offspring of radiation workers in Britain found no evidence of associations of leukaemia or lymphoma with PPI dose (Draper et al, 1997). The Committee on Medical Aspects of Radiation in the Environment (COMARE, 1996) has noted that 'we have not found any epidemiological study elsewhere to support Gardner's findings in Seascale in relation to preconception radiation effects'.

The results of Lord et al (1998) imply that an interaction between PPI and exposure to some leukaemogenic agent post-conception might be capable of explaining the Seascale cluster of childhood leukaemia. Previous work has shown that this explanation of the Seascale cluster is very unlikely to be true. First, the rate of production of the initiating events which would have to be induced by PPI in Seascale is at least 80 times greater than that predicted by conventional genetic processes (Wakeford et al, 1994). It is reasonable to expect that a mechanism of such a magnitude would have been detected before. Second, the strength of the interaction required to confine the PPI association to the less than 10% of the children of the Sellafield workforce born in Seascale implies an implausibly extreme supramultiplicative interaction between this putative other factor and PPI (Little et al, 1994). Third, excess incidence of childhood leukaemia has been observed in North Egremont, about 8 kilometres from Sellafield (Wakeford and Parker, 1996), and in West Thurso, near the Dounreay nuclear installation in Scotland (Kinlen et al, 1993). These leukaemia excesses cannot be explained by PPI (Urquhart et al, 1991; Wakeford and Parker, 1996). This is despite the fact that these excesses occur in communities containing large numbers of occupationally exposed men employed at the nearby nuclear installations. If PPI induces such a strong predisposition to childhood leukaemia that it can account for the Seascale cluster, it would be reasonable to expect that the leukaemogenic agent at work in North Egremont and West Thurso would also affect the significant numbers of supposedly susceptible children of occupationally exposed fathers living in these communities.

In summary, the observed associations in the study of Lord et al (1998) provide, at best, weak evidence for a causal effect of PPI on susceptibility to MNU-induced leukaemia and lymphoma yield in offspring of mice. Given the enormous doses of $^{239}$Pu and MNU administered, which are several orders of magnitude greater than occupational or environmental exposure levels, even if the associations observed by Lord et al (1998) are not due to chance or bias, it is unlikely that they are of any material relevance to man at occupational and environmental dose levels.

*MP Little*
*National Radiological Protection Board,*
*Chilton, Didcot,*
*Oxon OX11 0RQ, UK*

*MW Charles*
*School of Physics and Astronomy,*
*University of Birmingham, Edgbaston,*
*Birmingham B15 2TT, UK*

### REFERENCES

Beral V (1990) Leukaemia and nuclear installations. Occupational exposure of fathers to radiation may be the explanation (editorial). *Br Med J* **300**: 411–412

Committee on Medical Aspects of Radiation in the Environment (COMARE) (1996) Fourth Report. *The Incidence of Cancer and Leukaemia in Young People in the Vicinity of the Sellafield Site, West Cumbria: Further Studies and an Update of the Situation Since the Publication of the Report of the Black Advisory Group in 1984*. Department of Health: London

Draper GJ, Little MP, Sorahan T, Kinlen LJ, Bunch KJ, Conquest AJ, Kendall GM, Kneale GW, Lancashire RJ, Muirhead CR, O'Connor CM and Vincent TJ (1997) Cancer in the offspring of radiation workers: a record linkage study. *Br Med J* **315**: 1181–1188

Gardner MJ, Snee MP, Hall AJ, Powell CA, Downes S and Terrell JD (1990) Results of case-control study of leukaemia and lymphoma among young people near Sellafield nuclear plant in West Cumbria. *Br Med J* **300**: 423–429

Health and Safety Executive (HSE) (1993) *HSE Investigation of Leukaemia and Other Cancers in the Children of Male Workers at Sellafield*. Health and Safety Executive: Sudbury

Health and Safety Executive (HSE) (1994) *HSE Investigation of Leukaemia and Other Cancers in the Children of Male Workers at Sellafield: Review of Results Published in October 1993*. Health and Safety Executive: Sudbury

Kinlen LJ, O'Brien F, Clarke K, Balkwill A and Matthews F (1993) Rural population mixing and childhood leukaemia: effects of the North Sea oil industry in Scotland, including the area near Dounreay nuclear site. *Br Med J* **306**: 743–748; **307**: 91

Little MP, Wakeford R and Charles MW (1994) A comparison of the risks of leukaemia in the offspring of the Sellafield workforce born in Seascale and those born elsewhere in West Cumbria with the risks in the offspring of the Ontario and Scottish workforces and the Japanese bomb survivors. *J Radiol Prot* **14**: 187–201

Little MP, Wakeford R, Charles MW and Andersson M (1996) A comparison of the risks of leukaemia and non-Hodgkin's lymphoma in the first generation offspring ($F_1$) of the Danish Thorotrast patients with those observed in other studies of parental pre-conception irradiation. *J Radiol Prot* **16**: 25–36

Lord BI, Woolford LB, Wang L, Stones VA, McDonald D, Lorimore SA, Papworth D, Wright EG and Scott D (1998) Tumour induction by methyl-nitroso-urea following preconceptional paternal contamination with plutonium-239. *Br J Cancer* **78**: 301–311

Parker L, Craft A W, Smith J, Dickinson H, Wakeford R, Binks K, McElvenny D, Scott L and Slovak A (1993) Geographical distribution of preconceptional radiation doses to fathers employed at the Sellafield nuclear installation, West Cumbria. *Br Med J* **307**: 966–971

Selby PB (1990) Experimental induction of dominant mutations in mammals by ionizing radiations and chemicals. *Issues Rev Teratol* **5**: 181–253

Smith PG and Doll R (1982) Mortality among patients with ankylosing spondylitis after a single treatment course with x rays. *Br Med J* **284**: 449–460

Urquhart JD, Black RJ, Muirhead MJ, Sharp L, Maxwell M, Eden OB and Adams Jones D (1991) Case-control study of leukaemia and non-Hodgkin's lymphoma in children in Caithness near the Dounreay nuclear installation. *Br Med J* **302**: 687–692, 818

Wakeford R and Parker L (1996) Leukaemia and non-Hodgkin's lymphoma in young persons resident in small areas of West Cumbria in relation to paternal preconceptional irradiation. *Br J Cancer* **73**: 672–679

Wakeford R, Tawn EJ, McElvenny DM, Binks K, Scott LE and Parker L (1994) The Seascale childhood leukaemia cases – the mutation rates implied by paternal preconceptional radiation doses. *J Radiol Prot* **14**: 17–24

Weiss HA, Darby SC, Fearn T and Doll R (1995) Leukemia mortality after X-ray treatment for ankylosing spondylitis. *Radiat Res* **142**: 1–11

 *© Cancer Research Campaign 1999*

